# A CD146 FACS Protocol Enriches for Luminal Keratin 14/19 Double Positive Human Breast Progenitors

**DOI:** 10.1038/s41598-019-50903-9

**Published:** 2019-10-16

**Authors:** Ólöf Gerdur Ísberg, Jiyoung Kim, Agla J. Fridriksdottir, Mikkel Morsing, Vera Timmermans-Wielenga, Lone Rønnov-Jessen, Ole W. Petersen, René Villadsen

**Affiliations:** 1 Department of Cellular and Molecular Medicine and Novo Nordisk Foundation Center for Stem Cell Research, Faculty of Health and Medical Sciences, University of Copenhagen, Copenhagen, Denmark; 2 Biomedical Center, University of Iceland, Reykjavik, Iceland; 3 Division of Translational Cancer Research, Department of Laboratory Medicine, Lund University, Lund, Sweden; 4 Department of Pathology, Rigshospitalet, Copenhagen University Hospital, Copenhagen, Denmark; 5 Section for Cell Biology and Physiology, Department of Biology, Faculty of Science, University of Copenhagen, Copenhagen, Denmark

**Keywords:** Breast cancer, Cell biology, Adult stem cells

## Abstract

Human breast cancer is believed to arise in luminal progenitors within the normal breast. A subset of these are double positive (DP) for basal and luminal keratins and localizes to a putative stem cell zone within ducts. We here present a new protocol based on a combination of CD146 with CD117 and CD326 which provides an up to thirty fold enrichment of the DP cells. We show by expression profiling, colony formation, and morphogenesis that CD146^high^/CD117^high^/CD326^high^ DP cells belong to a luminal progenitor compartment. While these DP cells are located quite uniformly in ducts, with age a variant type of DP (vDP) cells, which is mainly CD146-negative, accumulates in lobules. Intriguingly, in specimens with *BRCA1* mutations known to predispose for cancer, higher frequencies of lobular vDP cells are observed. We propose that vDP cells are strong candidates for tracing the cellular origin of breast cancer.

## Introduction

Once fully developed, the breast belongs to a category of tissues and organs with relatively little cellular turnover. The breast epithelium contains two major lineages, and by far the majority of mitotic activity, when present, takes place within the luminal epithelial lineage^1^. This may explain why most breast cancers are caricatures of the cellular phenotypes found herein. We have previously investigated two major luminal cellular phenotypes in the human breast gland: Estrogen receptor α-positive (ERα^+^) cells and keratin 14-positive (K14^+^) cells of which the latter also express luminal-specific keratins (hereafter referred to as double positive, DP cells)^2–5^. Interestingly, similar phenotypes are found within the two most frequent subtypes of human breast cancer – the luminal subtype and basal-like subtype, respectively^6^. While we have devised a protocol for the prospective isolation and culture of the ERα^+ 2^, studying pure populations of DP cells has so far had to rely heavily on cloning of cultured cells with no or little prior enrichment^4,5^. However, assays based on collecting ducts and lobules under the microscope have strongly suggested that DP cells are indeed progenitors originating primarily from within a ductal stem cell zone^4^. Others have described DP cells as primarily a lobular characteristic^7,8^. In fact, there is increasing evidence for the existence of both ductal and lobular progenitor cells with different marker expression and differentiation potential^8–11^. The exact characterization of luminal progenitors has been complicated by the fact that these populations seem to be highly variable among individuals^10^. For instance, both the number and the distribution of progenitor cells appear to change with donor age or number of generations in culture^9,12^. In culture, we have previously enriched partially for DP cells based on CD117 and others have confirmed the progenitor properties of such populations^12,13^. However, CD117 is widely expressed in the luminal compartment of the adult breast^2,14^. Here, we enrich for DP cells by inclusion of CD146 in a FACS protocol and further show that the appearance of a CD146^neg^, variant DP (vDP) cell correlates with higher age and predisposition to cancer.

## Results

### DP and CD146-positive cells accumulate in ducts of the human normal breast gland

**Figure 1:**
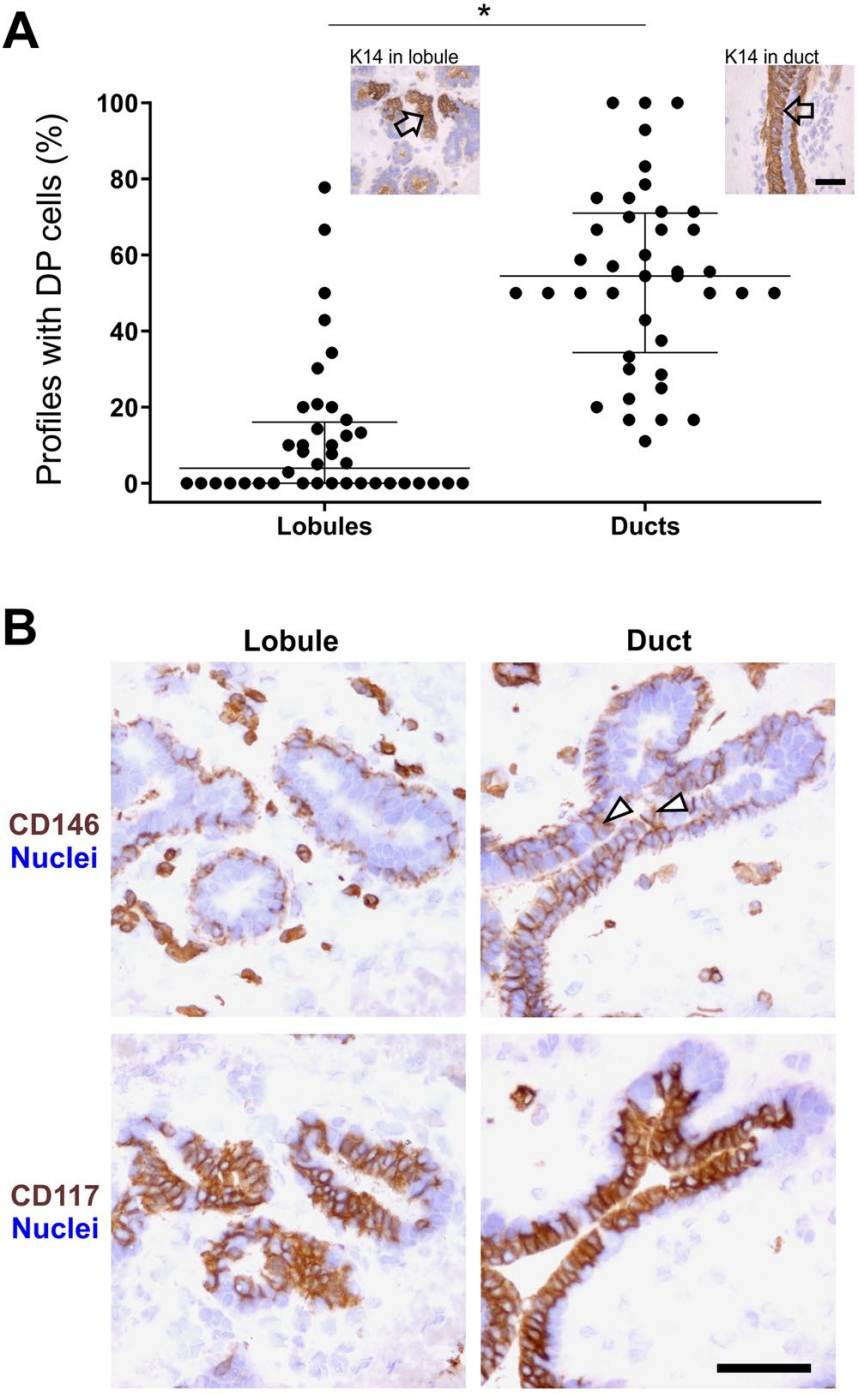
DP and CD146^+^ luminal cells accumulate in ducts of the human normal breast gland. (**A**) Dot plot comparing the proportion of structures containing DP cells shows that on average DP cells are more frequent in ducts. Image inserts show examples of DP cells marked by arrows in a lobule (left) and a duct (right) after immunoperoxidase stain. *p < 0.0001 tested by Mann-Whitney test (n = 40 biopsies, listed in Supplementary Table S1). Bar, 50 µm. (**B**) Representative immunoperoxidase stainings in serial sections demonstrating that CD146^+^ luminal cells are preferably present in ductal structures (upper panel), whereas CD117^+^ cells are generally present in both lobules and ducts (lower panel). Arrowheads mark examples of CD146^+^ cells in the luminal compartment. Bar, 50 µm.

We and others have previously demonstrated the presence of a stem cell zone rich in DP- or Aldehyde dehydrogenase-positive (ALDH^+^)  cells in the ducts of the human breast^4,15^. Here, based on immunohistochemistry and quantification of stained ductal and lobular profiles, in a sample of 40 human normal breast biopsies, we confirm a topographical accumulation of DP cells in ducts (Fig. 1A, Supplementary Fig. S1, Supplementary Tables S1 and S2). By applying candidates from our antibody repository^16^ in search for surface markers other than CD117 eligible for prospective isolation of DP cells we came across CD146, also referred to as melanoma cell adhesion molecule (MCAM). While CD146 in general is more widespread in stromal- and myoepithelial cells, in the luminal compartment, unlike CD117, CD146 is restricted mainly to the ducts (Fig. 1B, Supplementary Tables S3 and S4). Therefore, CD146 seemed promising as a more specific marker of DP cells than CD117.

### A CD146^high^/CD117^high^/CD326^high^ FACS protocol enriches for DP cells

**Figure 2:**
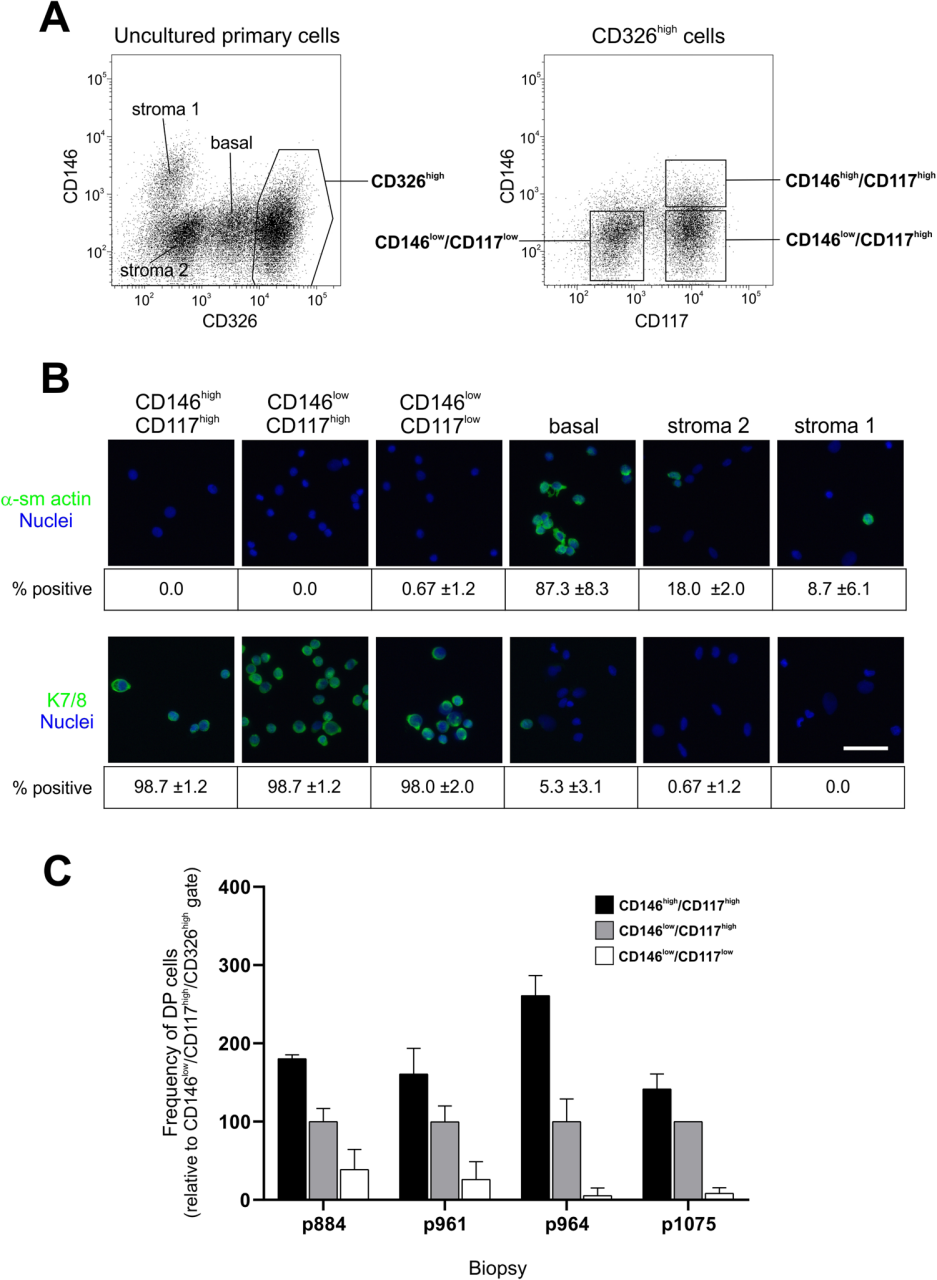
A CD146^high^/CD117^high^/CD326^high^ FACS protocol enriches for DP cells. (**A**) FACS diagrams showing uncultured primary cells gated into luminal CD326^high^ cells (left diagram, right frame) from which CD146^high^/CD117^high^, CD146^low^/CD117^high^ and CD146^low^/CD117^low^ populations were selected (right diagram). (**B**) Smears of sorted CD146^high^/CD117^high^, CD146^low^/CD117^high^, CD146^low^/CD117^low^, basal and stromal cells immunostained for α-sm actin and K7/8. Bar, 50 µm. The low frequencies of α-sm actin-positive cells in the luminal subpopulations are indicative of few, if any, contaminating basal cells. (**C**) Bar graph of the relative number of DP cells in FACS sorted populations showing a higher frequency of K14^+^/K19^+^ DP cells from the CD146^high^/CD117^high^ gate (black bars) compared to CD146^low^/CD117^high^ (grey bars) and CD146^low^/CD117^low^ cells (white bars) (p < 0.05 tested by ANOVA with Tukey HSD test, n = 4 biopsies). The frequency of CD146^low^/CD117^high^ DP cells was set at an arbitrary level of 100.

With the aim of enriching for DP cells we included CD146 in a previously described protocol used by us and others^2,4,17,18^ for isolation of CD326^high^ luminal cells and further stratified the cells according to CD117 expression (Fig. 2A). Thus, three subpopulations were sorted as CD146^high^/CD117^high^/CD326^high^, CD146^low^/CD117^high^/CD326^high^, and CD146^low^/CD117^low^/CD326^high^, in the following referred to as CD146^high^/CD117^high^¸ CD146^low^/CD117^high^ and CD146^low^/CD117^low^, respectively. The luminal identity of these cells was confirmed by the absence of α-smooth muscle actin (α-sm actin) and the presence of luminal keratin 7/8 in stainings of cellular smears (Fig. 2B). When comparing sorted luminal subpopulations with respect to the presence of DP cells in terms of K19/K14 double positivity the CD146^high^/CD117^high^ gate consistently came out as the one with the highest frequency of DP cells (Fig. 2C). The CD146^high^/CD117^high^ gate typically comprised 10% of the total CD326^high^ luminal population (ranging from 5 to 15%, data not shown). In summary, these results show that isolating a CD326^high^ luminal CD146^high^/CD117^high^ population enriches for DP cells.

### Characterization of CD146^high^/CD117^high^ cells

**Figure 3:**
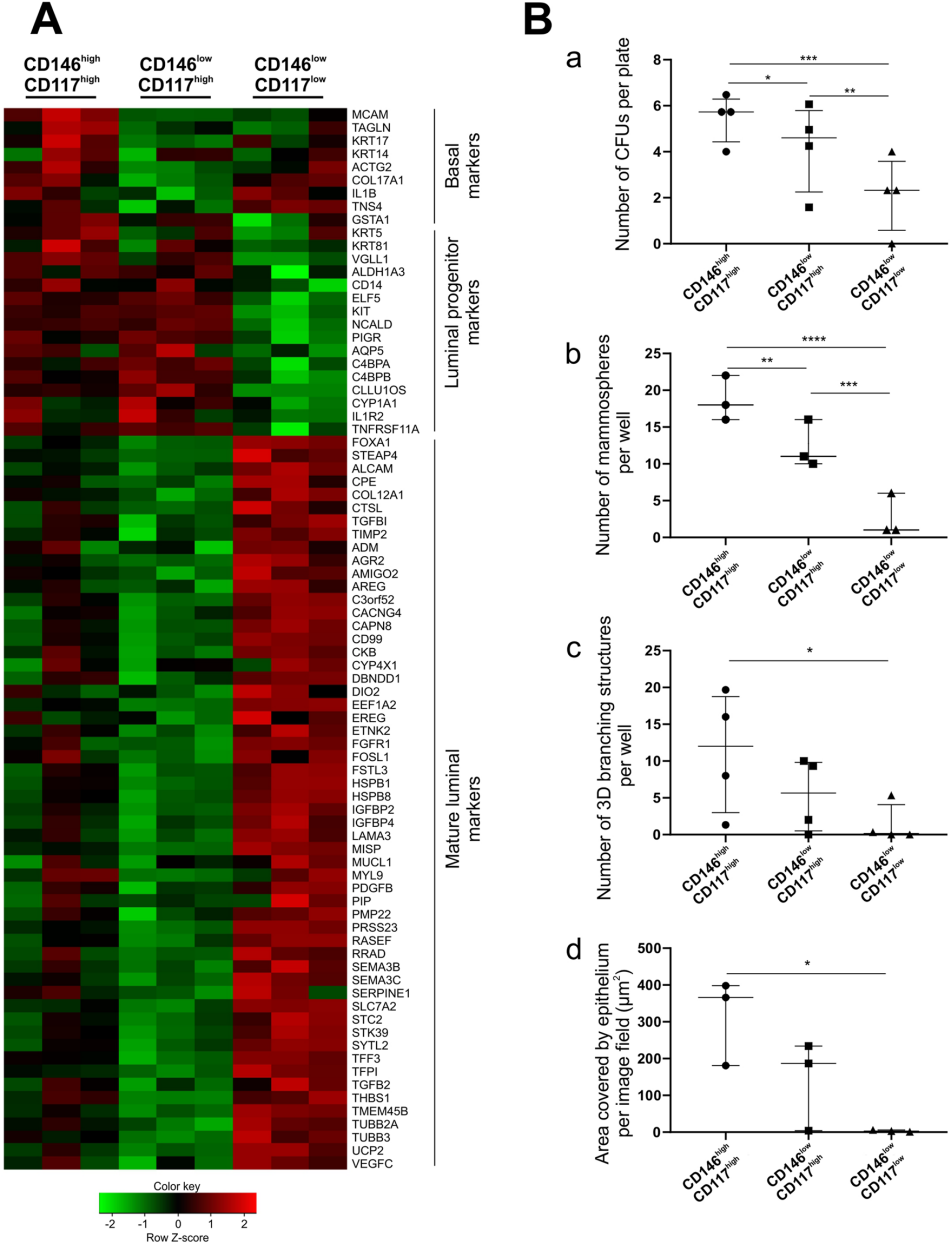
Characterization of CD146^high^/CD117^high^ cells. (**A**) Heatmap of selected DEGs in three luminal populations isolated by FACS. Genes are sorted according to lineage into basal, luminal progenitor and mature luminal markers. The RNA-Seq data demonstrate that the CD146^high^/CD117^high^ population differs from CD146^low^/CD117^high^ cells by a higher expression of basal markers, while CD146^low^/CD117^low^ cells have increased expression of mature luminal markers. Log_2_-scaled expression values are presented for each gene, and the color key indicate the Z-score (n = 3 biopsies). (**B**) Functional progenitor assays demonstrate that CD146^high^/CD117^high^ cells generally exhibit the highest level of activity. (a) In a colony forming unit (CFU) assay significantly more colonies were generated from CD146^high^/CD117^high^ cells compared to the other two populations (n = 4 biopsies). (b) In a mammosphere assay CD146^high^/CD117^high^ cells generated significantly more spheres than the other two populations (n = 3 biopsies). (c) In a 3D morphogenesis assay only CD146^high^/CD117^high^ cells developed significantly more branching structures than CD146^low^/CD117^low^ cells (n = 4 biopsies). (d) In a branching morphogenesis assay on fibroblast feeders only CD146^high^/CD117^high^ cells formed significantly more branching structures than CD146^low^/CD117^low^ cells (n = 3 biopsies). *p < 0.05, **p < 0.005, ***p < 0.0005, and ****p < 0.00005, tested by ANCOVA with Tukey’s HSD test. Each scatter dot plot is lined at median with interquartile range.

To compare CD146^high^/CD117^high^ cells with CD146^low^/CD117^high^and CD146^low^/CD117^low^ cells RNA sequencing (RNA-Seq) analysis was performed (Supplementary Tables S5 and S6) and differentially expressed genes (DEGs) among the three subpopulations were established by the NOISeq method (Supplementary Table S7). As revealed in a heatmap of selected DEGs, the CD146^high^/CD117^high^ cells expressed a number of basal/myoepithelial markers in addition to typical markers of progenitor cells (Fig. 3A). In this respect, it is worthwhile to emphasize that this expression concerns RNA only and not detectable protein. Thus, we have no reason to believe that it reflects contamination with myoepithelial cells per se. Rather, it may relate to what others have found in mice of some transient hybrid multipotent progenitors coexpressing basal and luminal markers^19^. Both CD146^high^/CD117^high^ as well as CD146^low^/CD117^high^ cells expressed relatively high levels of several luminal progenitor markers, including *KIT* and *TNFRSF11A*/RANK that have been correlated to progenitors accumulating in preneoplastic breast tissue of *BRCA1* mutation carriers^20,21^. By contrast, the CD146^low^/CD117^low^ cells remained mature luminal-like with higher levels of, for instance, *ALCAM* and *ESR1*/ERα (Fig. 3A). Application of gene ontology (GO) analysis as well as computerized pathway enrichment analysis using Kyoto encyclopedia of genes and genomes (KEGG) database further revealed cytoskeletal components and pathways of focal adhesions as most significantly different between CD146^high^/CD117^high^ and CD146^low^/CD117^high^ populations (Supplementary Table S8 and Supplementary Fig. S2).

To functionally assess the progenitor potential of the three sorted luminal subpopulations we analyzed their performance in the following assays: a colony forming unit (CFU) assay, a mammosphere assay and a three dimensional (3D) morphogenesis assay, which have all been implemented previously by us and others to analyze progenitor activity of breast epithelial cells^4,5,22,23^. In addition, we included a two dimensional (2D) feeder based morphogenesis assay which allows for analysis of branching structures upon epithelial-stromal interaction^24^. Based on these assays, the CD146^high^/CD117^high^ cells demonstrated the highest progenitor potential (Figs 3B and S3).

**Figure 4:**
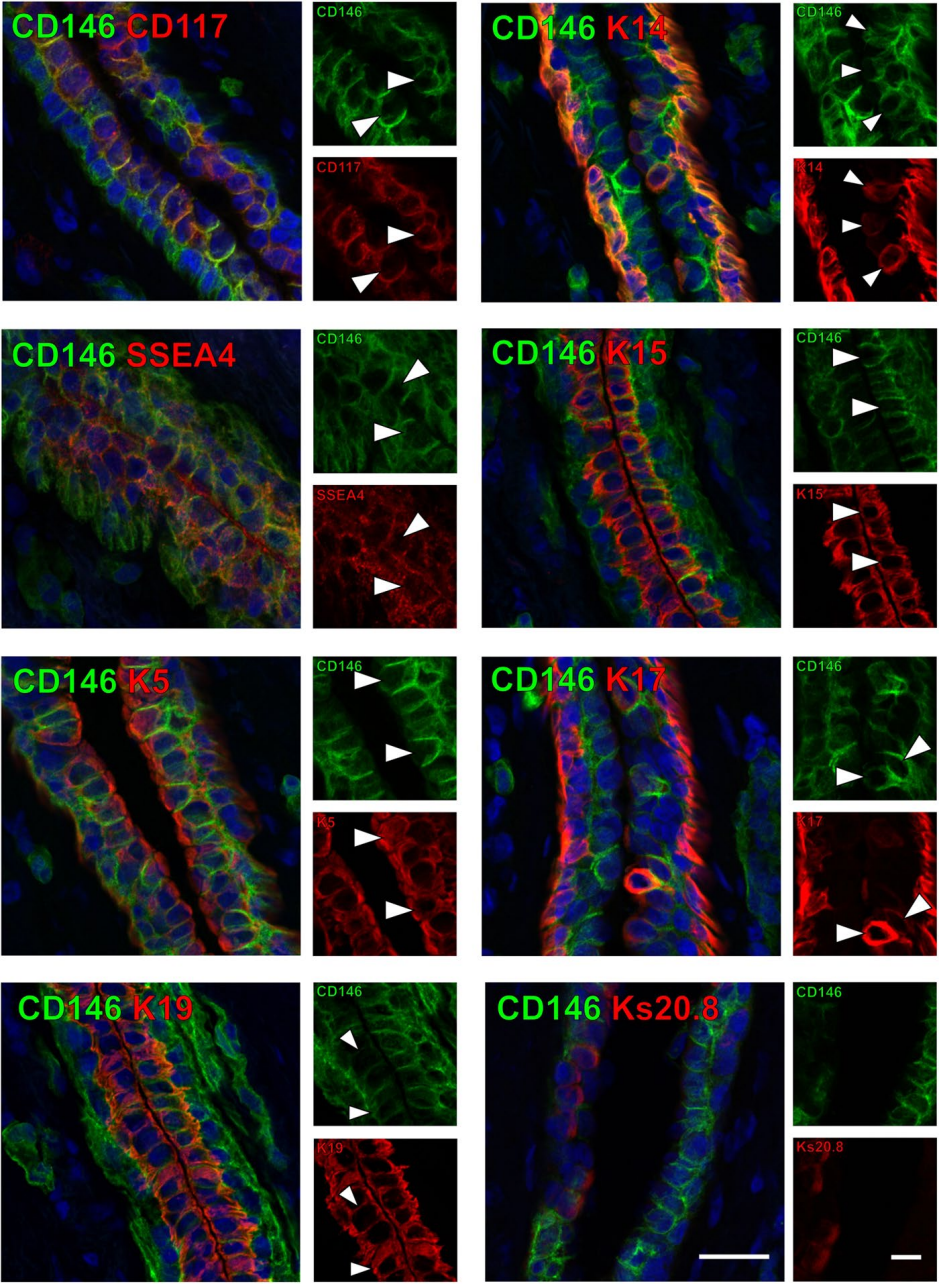
CD146^high^ luminal cells express with a number of progenitor markers. Immunofluorescent double stainings of CD146 (green) along with putative progenitor markers CD117, K14, SSEA4, K15, K5, K17 as well as K19 and Ks20.8 (all in red) in normal breast tissue sections. Nuclei are stained with DAPI (blue). Image subsets are shown in single color channels, and arrowheads mark examples of co-stained cells. While CD146^high^ cells co-express progenitor markers and K19, CD146^high^ and Ks20.8^+^ cells are generally confined to separate luminal compartments. Bar, 50 µm. Image subsets, bar, 20 µm.

In order to further characterize the luminal CD146^high^/CD117^high^ profile *in situ*, we performed multicolor immunohistochemistry that demonstrates correlation between CD146 and a number of putative progenitor markers in ductal structures (Fig. 4). As expected CD146-positive cells could be found to coexpress CD117 or K14. Furthermore, markers we previously described as being present in ductal stem cell zones, including SSEA4 as well as keratins 5, 15 and 17^4^, were co-expressed with CD146 in cells of the luminal compartment. By contrast, CD146 was generally not present in cells that were positive for mature luminal marker Ks20.8^2^.

Based on these findings, we conclude that inclusion of CD146 contributes a significant improvement over that of CD117/CD326 or CD326 only in attempts to prospectively isolate human breast DP cells with a progenitor profile.

### Variant DP cells accumulate in lobules with age and in tissue from BRCA1 mutation carriers

**Figure 5:**
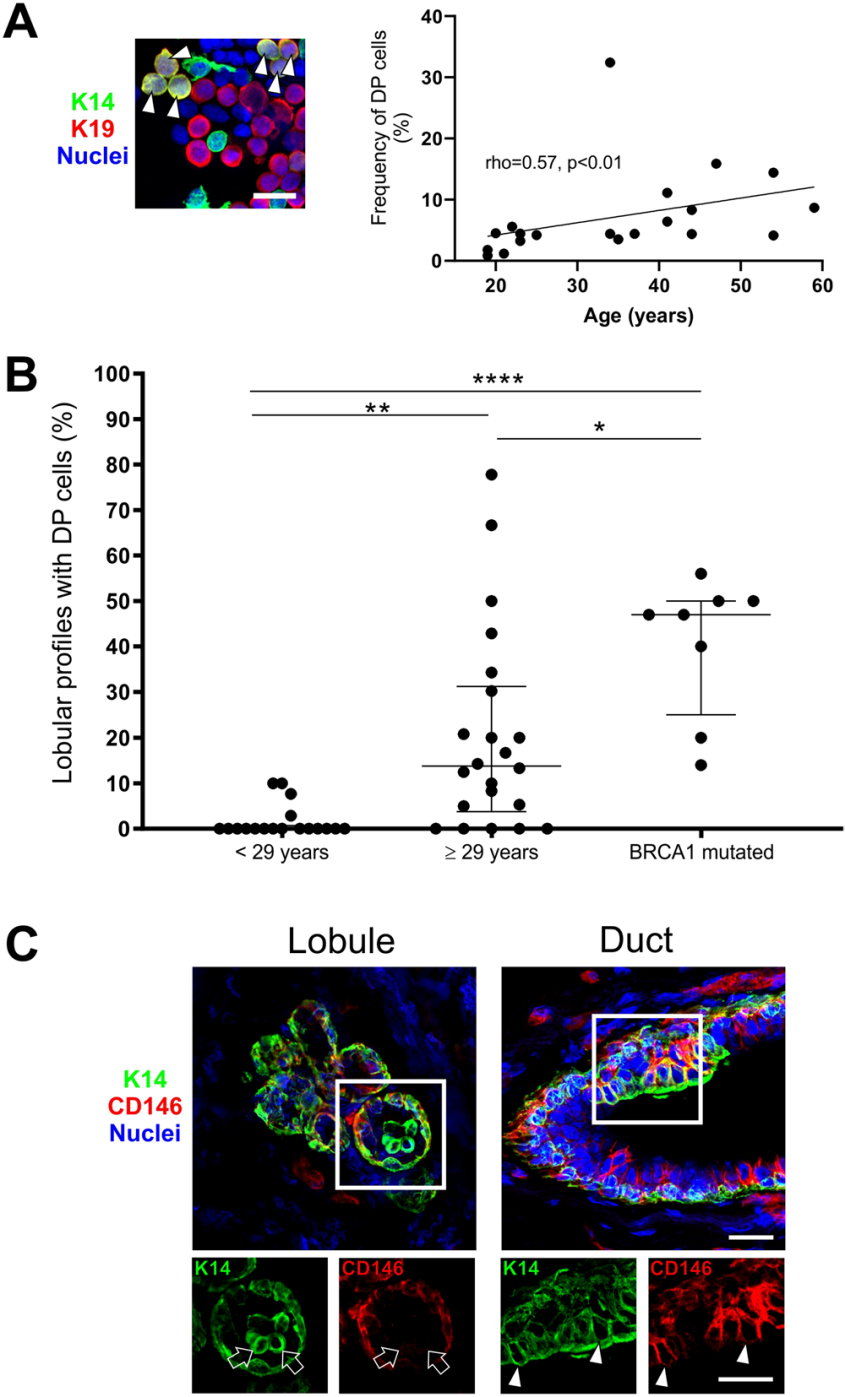
Variant DP cells accumulate in lobules with age and in tissue from *BRCA1* mutation carriers. (**A**) Immunofluorescent staining of crude smears with K14 (green), K19 (red) and nuclei (blue) (left image). Arrowheads mark DP cells. Bar, 20 µm. A positive correlation was found between age and the frequency of DP cells (right), analyzed by Spearman rank test (rho = 0.57, p < 0.01). (**B**) Dot plots of the proportion of lobular structures with DP cells in women <29 years (average age: 19.7 years, median: 19), ≥29 years (average age: 44.4 years, median: 43) and *BRCA1* mutation carriers (information about donor age not available). The age-selected data for lobules are derived from Supplementary Table S1, which are also included in Fig. 1A. The proportion of lobules containing DP cells is significantly higher in the older age group (20.5%) and in women with *BRCA1* mutations (40.5%) as compared to the younger age group (2.9%). *p < 0.05, **p < 0.005, and ****p < 0.000005 using ANOVA with Tukey’s HSD test. Each scatter dot plot is lined at median with interquartile range (<29 years: n = 18, ≥29 years: n = 22, BRCA1 mutated: n = 8). (**C**) Immunofluorescent staining demonstrating a segregation of DP and CD146^+^ cells in a lobule (left image) compared to a duct (right image) with CD146-expressing DP cells. K14 is shown in green, CD146 in red and nuclei in blue. Image subsets are shown in single color channels. Arrows indicate DP cells that are CD146^neg^ while arrowheads point to CD146^+^ DP cells. Bars, 25 µm.

**Figure 6:**
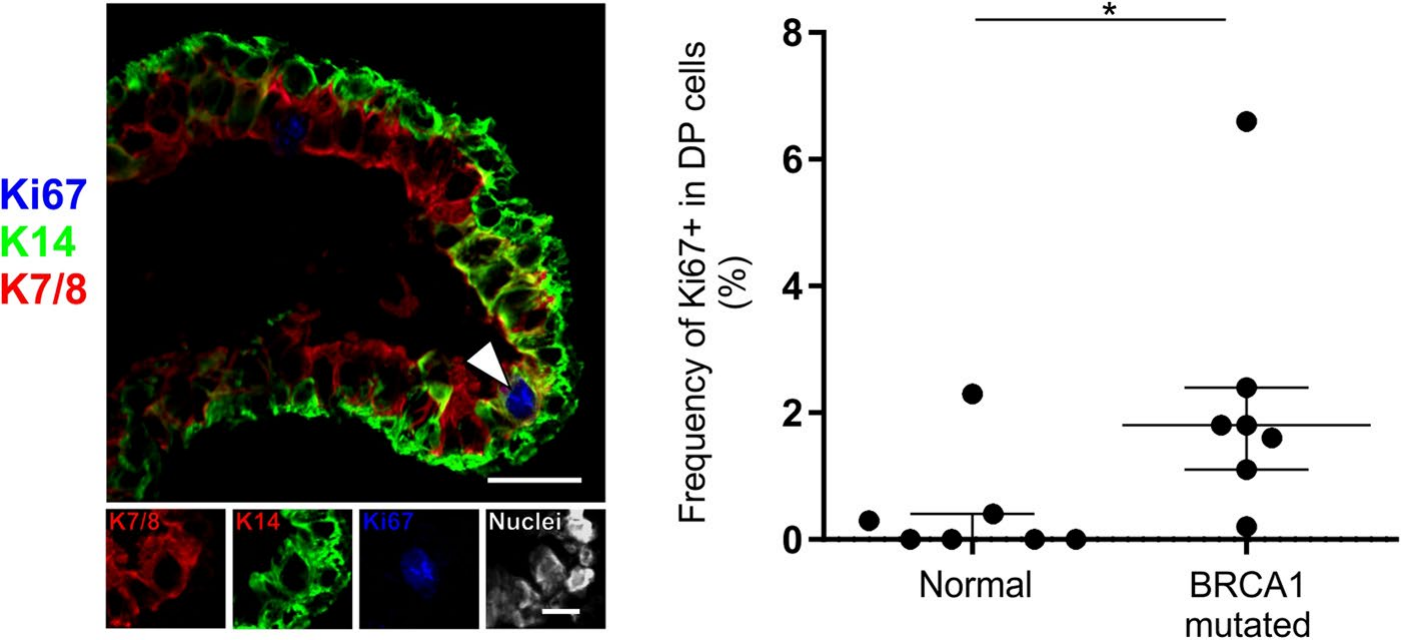
DP cells are more frequently cycling in tissue from BRCA1 mutation carriers. (**A**) Normal tissue from a woman with known BRCA1 mutation immunostained for K14 (green), cell cycle marker Ki67 (blue) and luminal keratin marker CAM5.2 (red). Arrowhead marks a Ki67^+^ DP cell. Bar, 25 µm. Lower panel image subsets are shown in single color channels, including DAPI nuclear stain (white). Bar: 10 µm. (**B**) Dot plot comparing the proportion of Ki67^+^ cells in the DP population between women with BRCA1 mutations and women with no known mutations. *p < 0.017 (normal: n = 7; BRCA1 mutated: n = 7) by Mann Whitney test.

We have previously reported that in culture the number of DP cells increases with the number of population doublings^12^. This prompted us to test whether the frequency of DP cells also *in situ* increases with age. Indeed, based on multicolor imaging of smears directly from biopsies we found an increase in relative frequency of DP cells with age (n = 20 samples, Fig. 5A and Supplementary Table S9). Keeping in mind that DP cells are already present in relatively high numbers in the ducts, it was not surprising that the age-related increase in DP numbers manifested itself in the lobules when comparing “young” (here defined as <29 years old with 2.9% lobules containing DP cells) and “old” (>29 years with 20.5% lobules containing DP cells) women (Fig. 5B). To analyze whether DP cells in lobules differ from DP cells in ducts we investigated a number of biopsies by immunofluorescent staining. As it turned out lobular DP cells were mostly CD146^neg^, and thus referred to here as variant DP (vDP cells) (Fig. 5C and Supplementary Table S10). This led us to speculate on a possible pathophysiological role of vDP cells in breast cancer evolution which is after all an age-related disease. To get a preliminary impression of this we examined a sample of breast tissue specimens from women with known mutations in the *BRCA1* gene and another sample of basal-like breast cancers with the majority of the neoplastic cells being DP. As the normal-derived samples from BRCA1 mutation carriers were completely anonymously donated, we could not make an exact age-matching of this material to that from presumed non-carriers. However, there is no reason to believe that the BRCA1-affected women were particularly old when undergoing mastectomy of the breast^21^. Irrespective of age, the tissue samples from *BRCA1* mutation carriers were characterized by having significantly more DP cells (40.5% lobules containing DP cells) (Fig. 5B). Furthermore, these were more active in terms of cell cycling (Fig. 6 and Supplementary Table S11). Both lobular DP cells from *BRCA1* mutation carriers and cancer associated DP were generally CD146^neg^ and as such similar to age related lobular vDP cells (Supplementary Tables S10 and S12).

Collectively, we conclude that CD146^high^/CD117^high^/CD326^high^ progenitors represent what could be referred to as the default luminal progenitor in the human breast and that additional vDP cells arise with age and under pathological conditions, suggesting that vDP cells represent precursor cells and their descendants in tissues predisposed to cancer development as well as in overt cancer.

## Discussion

In this study we present a novel protocol for enrichment of luminal progenitors harboring DP cells and provide evidence for their progenitor status. The preferential location of these progenitors within ductal zones is in line with our previous observation based on culturing cells from microcollected breast gland structures^4^. Apart from demonstrating an increased level of activity in culture-based progenitor assays, the CD146^high^/CD117^high^/CD326^high^ cells have a unique expression profile showing an increased level of basal/myoepithelial markers. This profile is reminiscent of the transient hybrid population that has been described during early development of the mouse mammary gland^19^, suggesting that the CD146^high^/CD117^high^/CD326^high^ compartment harbors an early luminal progenitor close to the apex of hierarchical differentiation tree. In support of this, others have recently published a study including single cell RNA profiling to evaluate the heterogeneity of ALDH^+^ luminal progenitors, in which the cluster with the highest levels of canonical markers of breast stemness also expressed high levels of CD146^10^. Both CD146^high^/CD117^high^ and CD146^low^/CD117^high^ cells express higher levels of several luminal progenitor markers compared to CD146^low^/CD117^low^ luminal cells, including TNFRSF11A/RANK. As with *KIT*/CD117 increased expression of RANK has been implicated with aberrant accumulation in preneoplastic breast epithelium of *BRCA1* mutation carriers, which in turn suggests that such cells could be a target for breast carcinogenesis^20,21^.

The age-related increase of DP cells reported here concurs with an earlier study in which culture based assays demonstrate age-dependent expansion of DP cells^12^. That DP cells tend to accumulate with age, and are more frequently present in lobular structures in women ≥29 years of age may be of significance with regard to carcinogenesis since the majority of breast cancers arises within terminal ductal lobular units^25^. Furthermore, the presence of CD146^neg^ or CD146^low^vDP cells in lobules implies that this population could be a different entity than its ductal counterpart expressing CD146. Recent data provided by our laboratory suggest that ducts and lobules do in fact contain basal stem cells or progenitors with different properties which may impact on the progeny at the respective anatomical regions^9^. Intriguingly, the vDP-like phenotype observed here in selected basal-like tumors is not incompatible with their origin in lobules.

BRCA1 mutation carriers have a profound increased lifetime breast cancer risk^26^. Lim *et al*. reported that the breast glands of BRCA1 mutation carriers have increased numbers of luminal progenitors, suggesting that this population contains the cell of origin for subsequent tumor formation^20^. While here we cannot correlate the data of the BRCA sample with age, indeed, irrespective of age, the frequency of DP cells is higher among BRCA1 mutation carriers. DP cells of BRCA1 mutation carriers are also more frequently cycling compared to non-carriers as demonstrated by Ki67 staining, which supports that these women may host luminal progenitors at an aberrant frequency. An increased proliferative state could also imply an increased risk of accumulating transforming mutations in these cells. Furthermore, just as is generally the case for sporadic basal-like tumors, breast carcinomas derived from BRCA1 mutation carriers are more proliferative when compared to ERα^+^ tumors^27,28^.

In conclusion, we suggest that the luminal progenitor compartment containing DP cells is key for understanding the cellular origin of breast cancer.

## Materials and Methods

### Human breast tissue samples

Normal breast biopsies were acquired from healthy women undergoing reduction mammoplasty for cosmetic reasons at Capio CFR Hospitaler, Lyngby or Hellerup, Denmark. All donors were informed before surgery and agreed by written consent to donate tissue. The specimen donor’s personal information was confidential and protected except the date and year of birth. In some cases only the year of birth was available. Material from some of the biopsies have been used previously in several studies^2,9,16^; however, picking the sample material was done randomly and all relevant tissue processing were done specifically for this study which has not been previously published. Normal breast tissue specimens from *BRCA1* mutation carriers as well as breast carcinomas were obtained completely anonymously from patients at the State University Hospital, Copenhagen. The storage and use of human material has been reviewed and approved by the Regional Scientific Ethical Committees (H-2-2011-052 and H-3-2010-095) and the Danish Data Protection Agency (2011-41-6722) and has been handled according to established guidelines in subsequent experiments. In total, normal-derived samples from 80 women were included of which 10 were known BRCA1 mutation carriers. Of the biopsies that were used for flow cytometry four were immunostained, three biopsies were submitted to RNA expression profiling and nine biopsies were included in cell culture assays. 23 ERα-negative breast carcinomas were selected from a sample material of 150 invasive breast carcinomas.

### Immunostaining

*Immunosmears:* The 20 biopsies included for staining of crude smears were picked randomly among material used for other routine analyses in the lab. Isolation of breast epithelial organoids was performed as previously described^29^. Primary epithelial organoids were dissociated into single cells with 0.25% trypsin and 1 mmol/L EDTA. Cells were suspended in PBS with 10% goat serum, dripped onto glass slides with a pipette and air dried before fixation in either ice cold methanol for 5 minutes or in 3.7% formaldehyde (Merck, Darmstadt, Germany) followed by permeabilization with Triton X-100 detergent (Sigma-Aldrich). Smears were incubated with primary antibodies for at least 2 hours before incubation with isotype-specific secondary fluorescent antibodies for 30 minutes (Supplementary Table S13). Double positive (DP) cells were detected by co-expression of K14/K19. Additionally, crude smears from some biopsies were also analyzed by co-expression of K14/K7/8 (using CAM5.2 that detects keratins 7 and 8^30^). K7 is more widely expressed in the luminal compartment compared to K19^9^. Cells sorted by FACS were spun down, dripped onto glass slides and fixed as described above. Quantification of DP cells among sorted cells was done by quantifying 3 × 100 cells for each population (n = 4 biopsies). Analysis of the presence of basal cells and luminal cells in sorted cells was performed with staining for α-sm actin and CAM5.2, respectively. For each population 3 × 100 cells were quantified. Nuclei were stained with DAPI (Life Technologies, Thermo Fisher Scientific). *Immunohistochemistry:* Snap frozen tissue specimens were cut at 6 µm on a cryostat (Microm HM560, Axlab, Denmark) and were subsequently fixed either in methanol or formaldehyde as described for immunosmears. For peroxidase stainings, Ultravision ONE Detection System was utilized according to the manufacturer’s instructions (Labvision, Thermo Fisher Scientific). Nuclei were stained with hematoxylin. For immunofluorescence the specimens were incubated for 2 hours with primary antibody followed by incubation with secondary isotype-specific Alexa Fluor antibodies, as described for stainings of smears. Nuclei were stained with DAPI. Antibodies and dilutions are listed in Supplementary Table S13.

### Microscopy

Routine analysis of brightfield and fluorescent samples were performed on a Leica DM550B microscope. Peroxidase images were acquired using this microscope equipped with a DFC550 digital camera (Leica). Fluorescent images presented in the figures were acquired using a confocal microscope (Zeiss LSM 700) located at the Core Facility for Integrated Microscopy, Faculty of Health and Medical Sciences, University of Copenhagen. Phase contrast images were acquired using a Leica DM IL equipped with a MC170 HD digital camera. For image acquisition at low magnification a Leica MDG41 equipped with a MC170 HD digital camera was used.

### Quantification and statistical analysis

Quantification of the frequency of structures containing luminal K14^+^ cells were done under the microscope based on sections stained by immunoperoxidase. A lobule or duct with one or more positive cells present was scored as positive. Evaluation of the distribution of CD146- and CD117-expressing luminal cells were performed on serial sections stained by immunoperoxidase. Quantification of the frequency of K14/K19 and K14/K7/8 DP cells on smears were performed either directly under the microscope utilizing filters that visualize Alexa Fluor 488, Alexa Fluor 568 or both simultaneously or on images that were acquired and digitally stored. Quantification of the frequency of K14/K7/8 DP cells in ducts and lobules (Table S2) were performed on digitally stored images. In both cases areas of interest was located based on nuclear DAPI stain to avoid any bias in selection. A TDLU consists of the most distal part of the ductal-lobular system and include the collection of lobular ductules/acini branching from the intralobular terminal duct, as well as the connecting extralobular terminal duct^31^ (Fig. S1). For evaluation and quantification of DP cells in lobules, intralobular ducts were regarded as a ductal segment to distinguish between ductules/acini and the corresponding duct when visually distinguishable – either by histology or by the presence of distinct K14^+^/K7/8^−^ basal cells. Quantification of the proportion of DP cells expressing Ki67 was done using a confocal microscope by first assessing the number DP cells in a structure before evaluating Ki67 expression by image acquisition (Supplementary Table S11). Generally, several sections from a biopsy sample were evaluated. All statistical analyses were performed using the computing program R (Version 3.5.1) and graphs were drawn either in R or the graphic pad Prism (version 8.0) and mentioned in figure- and table legends where statistics was used. In Denmark the average age of primiparous women is 29 years (Danmarks Statistik 2017, www.statistikbanken.dk/FOD11), which was used to divide women into two age-groups (Fig. 5B).

### FACS

Primary breast organoids were dissociated into single cells as described for immunosmears. Viable cell suspensions were filtered through a 100 µm filter and resuspended in HEPES buffer supplemented with 0.5% BSA (bovine fraction V; Sigma-Aldrich) and 2 mM EDTA, and incubated for 30 min at 4 °C with antibodies CD146 (1:20, clone P1H12, Alexa Fluor (AF) 647 conjugate, BD Biosciences), CD117 (1:20, clone 104D2, PE conjugate, BD Biosciences) and CD326 (1:20, clone 9C4, AF488 conjugate, BioLegend) (See Supplementary Table S13). Controls were incubated without antibodies and in selected experiments as Fluorescence Minus One (FMO) controls omitting CD146 antibody. Following incubation the cells were washed twice in HEPES/BSA/EDTA buffer and filtered through a 10 or 20 µm filter cup (Filcons). For dead cell discrimination, 1 µg/mL propidium iodide (Invitrogen) was added prior to analysis in a flow cytometer (FACSAria II or FACSFusion, BD Biosciences). When sorting into 96 well plates the instrument’s automated cell deposition unit (ACDU) and single cell sorting parameters were utilized.

### RNA-Sequencing

Total RNA from CD146^high^/CD117^high^, CD146^low^/CD117^high^ and CD146^low^/CD117^low^ CD326^high^ luminal cells sorted from three different biopsies was isolated using 500 µL TRIzol® (Invitrogen) and a spin-column method (Zymo research). Library construction, sequencing process and the data analysis were conducted by Beijing Genomics Institute, as described previously^9^. In short, cDNA library of the enriched mRNA was fragmented into 200 bps and sequencing adaptors were ligated and amplified before quality control with a Agilient 2100 bioanalyzer. The libraries were sequenced on a HiSeq2000 sequencer (Illumina), 50 bp high output to average depth of 10 million reads. The summary of RNASeq data is included in Supplementary Table S5 and a detailed list of genes in Supplementary Table S6. Readout alignment was performed by SOAP (version 2.21) and gene expression was quantified by RPKM algorithm. Differently expressed genes among groups were identified by the NOISeq method using a cutoff value as fold difference higher than 2 between two groups with probability higher than 0.8^32^. The selection of basal-, luminal progenitor- and mature luminal markers shown in the heatmap in Fig. 3A was based on previously published expression data^2,20,33^. GO and KEGG databases were used for gene ontology enrichment analysis and pathway enrichment analysis, respectively.

### Cell culture

All cell cultures were incubated in a humidified incubation chamber in 5% CO_2_ at 37 °C.

*CFU assay:* Each of the CD146^high^/CD117^high^, CD146^low^/CD117^high^ and CD146^low^/CD117^low^ subpopulations were sorted directly into a 96 well plate precoated with collagen I (Corning) as 10 cells per well in the presence of 100 µL FAD2 medium (DMEM, high glucose, no calcium (Life Technologies) 3:1 v/v with 2 mM glutamine, 0.5 µg/mL hydrocortisone (Sigma-Aldrich), 5 µg/mL insulin (Sigma-Aldrich), 10 ng/mL cholera toxin (Sigma-Aldrich), 10 ng/mL EGF (Peprotech), 1.8 × 10^−4^ M adenine, 10 µM Y-27632 (Y0503, Sigma-Aldrich) and 5% FCS (Sigma-Aldrich)) as described in^2^. Cultures were monitored for 14 days before fixation and hematoxylin staining. Proliferating clonal islets were determined under a microscope as colonies consisting of more than 25 cells. The sample material was derived from four different biopsies. *Mammosphere assay:* Sorted cells were counted and plated in triplicates in ultra-low attachment 24-well plates (Costar) with 5,000 cells/well in mammary epithelial growth medium (MEGM, MEBM (Lonza) supplemented with 2% B27 (Invitrogen), 20 ng/mL EGF (Peprotech), 20 ng/mL bFGF (Peprotech), and 4 µg/mL heparin (Sigma)). Cultures were monitored for 14 days before final evaluation was performed by counting mammospheres ≥80 µm in diameter. The sample material was derived from three different biopsies. *3D morphogenesis assay:* Sorted cells were counted and 20,000 cells per well were embedded in triplicates in 300 µL Matrigel (BD Biosciences) on top of a primary epithelial feeder overlayed by 250 µL Matrigel in 24 well plates. The cells were cultured in F12 medium (Ham’s F12 (Invitrogen) supplemented with 2 mM glutamine, 5 µg/mL insulin, 1 µg/mL hydrocortisone, 0.1 µg/mL cholera toxin, 10 ng/mL EGF and 5% FCS). After 21 days branching structures were quantified under a phase contrast microscope. The sample material was derived from four different biopsies. *Branching morphogenesis assay with epithelial-stromal interaction:* Sorted cells were seeded in triplicates of 5,000 cells on confluent human CD105^high^/CD26^low^ fibroblast feeders in T12.5 culture flasks. This co-culture condition was previously established with modified breastoid base medium without HEPES (DMEM/F-12, 1:1), 1 μg/mL hydrocortisone (Sigma-Aldrich), 9 μg/mL insulin (Sigma-Aldrich), 5 μg/mL transferrin (Sigma-Aldrich), 5.2 ng/mL Na-Selenite (BD Industries), 100 μM ethanolamine (Sigma-Aldrich), 20 ng/mL bFGF, 5 nM amphiregulin (R&D Systems), with the addition of 10 μM Y-27632 (Axon Medchem), 1.8 × 10^−4^ M adenine (Sigma Aldrich) and the serum replacement B27 (20 μL/mL, Life Technologies)^24^. After 14 days cultures were fixed and stained for K19 by immunoperoxidase. After image acquisition stained epithelial structures were analyzed and quantified (ImageJ version 1.49 t) as described previously^24^. Briefly, epithelial structures were segmented followed by analysis of the number of structures and the total area using the plugin Analyse Particles. The sample material was derived from three different biopsies.

## Supplementary information

Suppplementary Information

Supplementary Table S5

Supplementary Table S6

Supplementary Table S7

Supplementary Table S8

## References

[1] Clarke RB, Howell A, Potten CS, Anderson E (1997). Dissociation between steroid receptor expression and cell proliferation in the human breast. Cancer Res..

[2] Fridriksdottir AJ (2015). Propagation of oestrogen receptor-positive and oestrogen-responsive normal human breast cells in culture. Nat. Commun..

[3] Hopkinson, B. M. *et al*. Establishment of a normal-derived estrogen receptor-positive cell line comparable to the prevailing human breast cancer subtype. *Oncotarget* **8** (2017). 10.18632/oncotarget.14554 PMC5354682 28076334

[4] Villadsen R (2007). Evidence for a stem cell hierarchy in the adult human breast. J. Cell Biol..

[5] Gudjonsson T (2002). Isolation, immortalization, and characterization of a human breast epithelial cell line with stem cell properties. Genes Dev..

[6] Alshareeda AT (2013). Characteristics of basal cytokeratin expression in breast cancer. Breast Cancer Res. Treat..

[7] Santagata S (2014). Taxonomy of breast cancer based on normal cell phenotype predicts outcome. J Clin Invest.

[8] Honeth G (2015). Models of Breast Morphogenesis Based on Localization of Stem Cells in the Developing Mammary Lobule. Stem Cell Reports.

[9] Fridriksdottir AJ (2017). Proof of region-specific multipotent progenitors in human breast epithelia. Proc Natl Acad Sci USA.

[10] Colacino JA (2018). Heterogeneity of Human Breast Stem and Progenitor Cells as Revealed by Transcriptional Profiling. Stem Cell Reports.

[11] Arendt LM (2014). Anatomical localization of progenitor cells in human breast tissue reveals enrichment of uncommitted cells within immature lobules. Breast Cancer Res..

[12] Garbe JC (2012). Accumulation of multipotent progenitors with a basal differentiation bias during aging of human mammary epithelia. Cancer Res..

[13] Britschgi A (2017). The Hippo kinases LATS1 and 2 control human breast cell fate via crosstalk with ERα. Nature.

[14] Kim J, Villadsen R (2018). Expression of Luminal Progenitor Marker CD117 in the Human Breast Gland. J. Histochem. Cytochem..

[15] Honeth G (2014). Aldehyde dehydrogenase and estrogen receptor define a hierarchy of cellular differentiation in the normal human mammary epithelium. Breast Cancer Res..

[16] Balk-Møller E (2014). A marker of endocrine receptor-positive cells, CEACAM6, is shared by two major classes of breast cancer: Luminal and HER2-enriched. Am. J. Pathol..

[17] Eirew P (2008). A method for quantifying normal human mammary epithelial stem cells with *in vivo* regenerative ability. Nat. Med..

[18] Stingl J, Eaves CJ, Kuusk U, Emerman JT (1998). Phenotypic and functional characterization *in vitro* of a multipotent epithelial cell present in the normal adult human breast. Differentiation..

[19] Wuidart A (2018). Early lineage segregation of multipotent embryonic mammary gland progenitors. Nat. Cell Biol..

[20] Lim E (2009). Aberrant luminal progenitors as the candidate target population for basal tumor development in BRCA1 mutation carriers. Nat. Med..

[21] Nolan E (2016). RANK ligand as a potential target for breast cancer prevention in BRCA1-mutation carriers. Nat. Med..

[22] Clayton H, Titley I, Vivanco M (2004). Growth and differentiation of progenitor/stem cells derived from the human mammary gland. Exp. Cell Res..

[23] Dontu G (2003). *In vitro* propagation and transcriptional profiling of human mammary stem/progenitor cells. Genes Dev..

[24] Morsing M (2016). Evidence of two distinct functionally specialized fibroblast lineages in breast stroma. Breast Cancer Res..

[25] Tabár L (2014). A Proposal to Unify the Classification of Breast and Prostate Cancers Based on the Anatomic Site of Cancer Origin and on Long-term Patient Outcome. Breast Cancer Basic Clin. Res..

[26] Kuchenbaecker KB (2017). Risks of Breast, Ovarian, and Contralateral Breast Cancer for BRCA1 and BRCA2 Mutation Carriers. JAMA.

[27] Reis-Filho JS, Tutt ANJ (2007). Triple negative tumours: a critical review. Histopathology.

[28] Hassanein M (2013). Prediction of BRCA1 germ-line mutation status in patients with breast cancer using histoprognosis grade, MS110, Lys27H3, vimentin, and KI67. Pathobiology.

[29] Rønnov-Jessen L, Petersen OW (1993). Induction of alpha-smooth muscle actin by transforming growth factor-beta 1 in quiescent human breast gland fibroblasts. Implications for myofibroblast generation in breast neoplasia. Lab. Invest..

[30] Hsu J-D, Yao C-C, Han L-W, Han C-P (2010). CAM5.2 Is Not Identical to Cytokeratins 8 and 18. Am. J. Clin. Pathol..

[31] Rønnov-Jessen L, Petersen OW, Bissell MJ (1996). Cellular changes involved in conversion of normal to malignant breast: importance of the stromal reaction. Physiol. Rev..

[32] Tarazona S, García-Alcalde F, Dopazo J, Ferrer A, Conesa A (2011). Differential expression in RNA-seq: a matter of depth. Genome Res..

[33] Lim E (2010). Transcriptome analyses of mouse and human mammary cell subpopulations reveal multiple conserved genes and pathways. Breast Cancer Res..

