# Publisher Correction: A CD146 FACS Protocol Enriches for Luminal Keratin 14/19 Double Positive Human Breast Progenitors

**DOI:** 10.1038/s41598-020-60887-6

**Published:** 2020-03-11

**Authors:** Ólöf Gerdur Ísberg, Jiyoung Kim, Agla J. Fridriksdottir, Mikkel Morsing, Vera Timmermans-Wielenga, Lone Rønnov -Jessen, Ole W. Petersen, René Villadsen

**Affiliations:** 1 Department of Cellular and Molecular Medicine and Novo Nordisk Foundation Center for Stem Cell Research, Faculty of Health and Medical Sciences, University of Copenhagen, Copenhagen, Denmark; 2 Biomedical Center, University of Iceland, Reykjavik, Iceland; 3 Division of Translational Cancer Research, Department of Laboratory Medicine, Lund University, Lund, Sweden; 4 Department of Pathology, Rigshospitalet, Copenhagen University Hospital, Copenhagen, Denmark; 5 Section for Cell Biology and Physiology, Department of Biology, Faculty of Science, University of Copenhagen, Copenhagen, Denmark

Correction to: *Scientific Reports* 10.1038/s41598-019-50903-9, published online 16 October 2019

In the original version of this Article, the author Ólöf Gerdur Ísberg was incorrectly indexed. This error has now been corrected.

