# Supplementary material for: A CD146 FACS Protocol Enriches for Luminal Keratin 14/19 Double Positive Human Breast Progenitors
Source: Sci Rep. 2019 Oct 16;9:14843. doi: 10.1038/s41598-019-50903-9 (PMC6795797; doi:10.1038/s41598-019-50903-9)
Supplement: Supplementary file 1 — Suppplementary Information [file 41598_2019_50903_MOESM1_ESM.pdf]

# A CD146 FACS PROTOCOL ENRICHES FOR LUMINAL KERATIN 14/19 DOUBLE POSITIVE HUMAN BREAST PROGENITORS

Ólöf Gerdur Ísberg, Jiyoung Kim, Agla J. Fridriksdottir, Mikkel Morsing, Vera Timmermans-Wielenga, Lone Rønnov-Jessen, Ole William Petersen & René Villadsen

## Supplementary Information:

Supplementary Figures

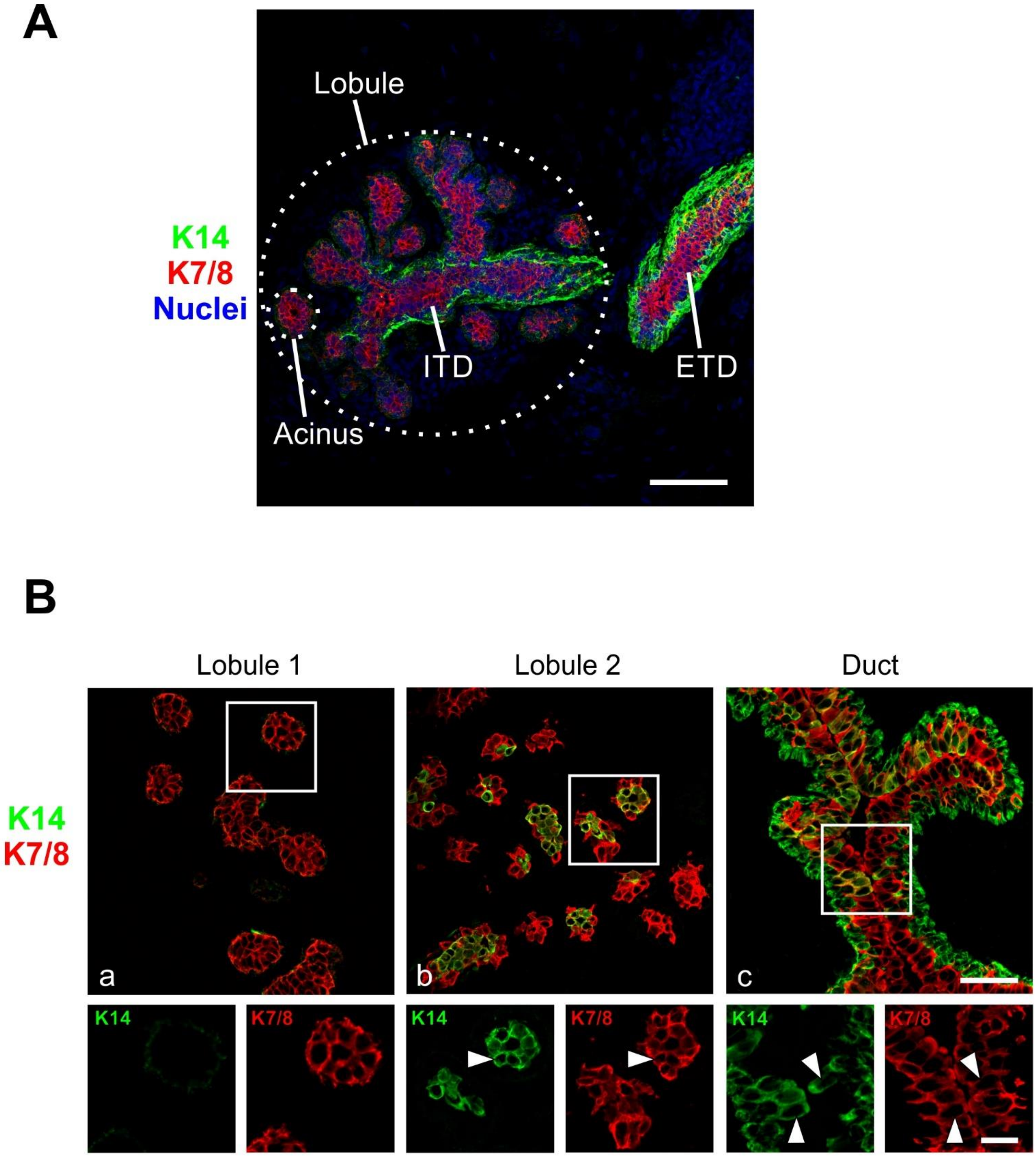

**Supplementary Figure S1**. Keratin 14$^+$ DP cells can be found in distinct profiles of the human breast gland. (A) Immunofluorescent staining of human breast gland by K14 (green) and K7/8 (red). Nuclei are stained in blue. The adult human breast gland can be divided into lobular and ductal structures. A lobule consists of an intralobular duct (ITD) that braches into a number of ductules/acini. The ITD is connected to the extralobular duct (ETD) outside the lobular structure. Note that the basal cells of the ducts generally strongly express K14, while the myoepithelium in the acini often shows diminished staining. Bar, 100 µm. (B) Immunofluorescent staining performed as above demonstrating examples of the distribution of DP cells in lobules (a, b) and ducts (c). Image subsets are marked and shown in single color channels, and arrowheads mark examples of DP cells. While K14$^+$ DP cells are most prominently found in ducts, occasionally DP cells are observed in lobular acini. Bar, 50 µm. Image subsets, bar, 20 µm.

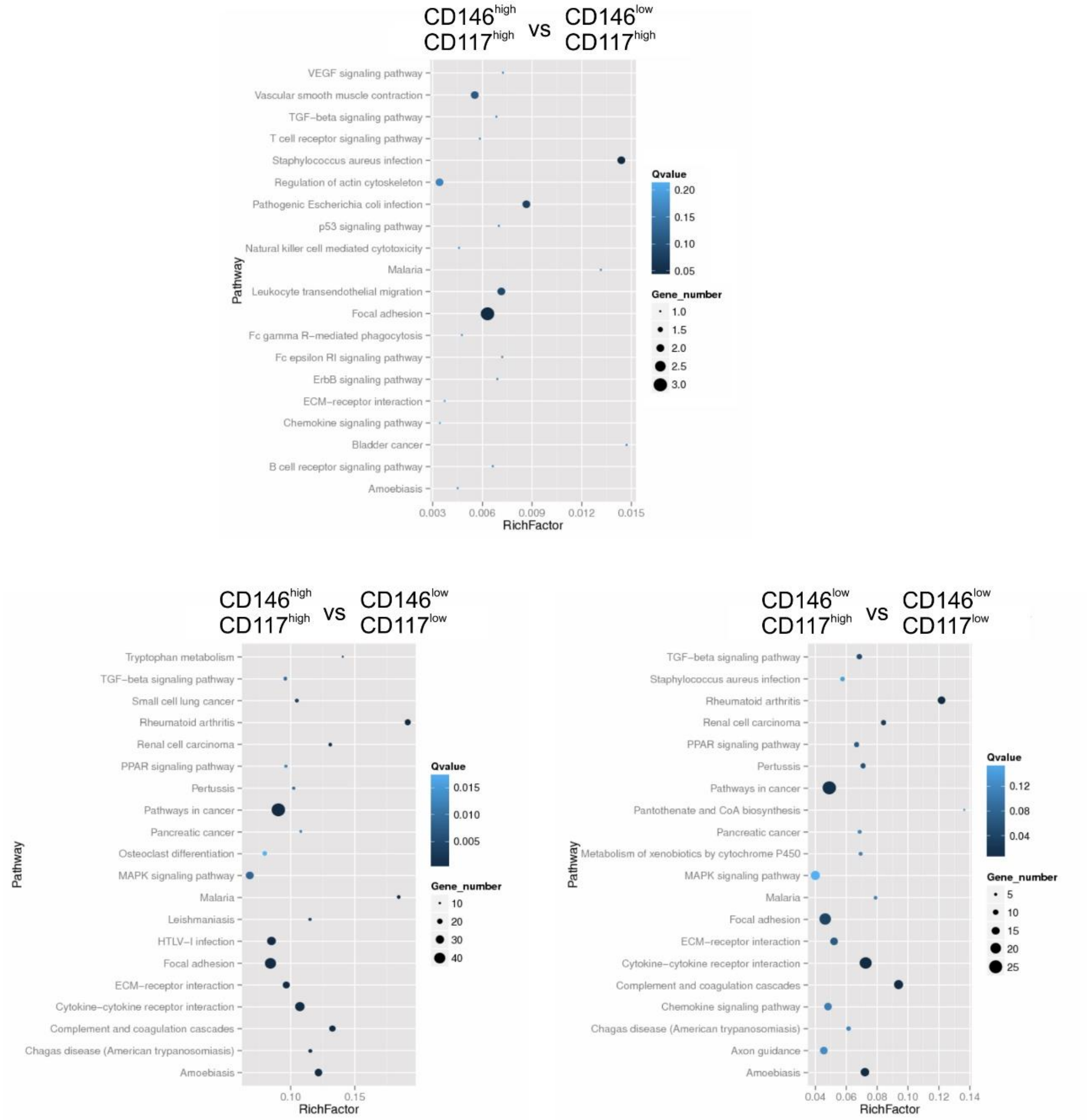


**Supplementary Figure S2**. Classification of gene expression patterns from RNA-Seq data reveals subtle differences between the sorted epithelial subpopulations. Scatter plots of KEGG pathway enrichment statistics, comparing CD146$^{high}$/CD117$^{high}$ with CD146$^{low}$/CD117$^{high}$ and CD146$^{low}$/CD117$^{low}$ as well as CD146$^{low}$/CD117$^{high}$ with CD146$^{low}$/CD117$^{low}$. Rich Factor indicates the ratio between DEGs and the number of genes annotated in the given pathway. Q Value indicates adjusted p value depicting significant enrichment (Q value < 0.05, navy blue).

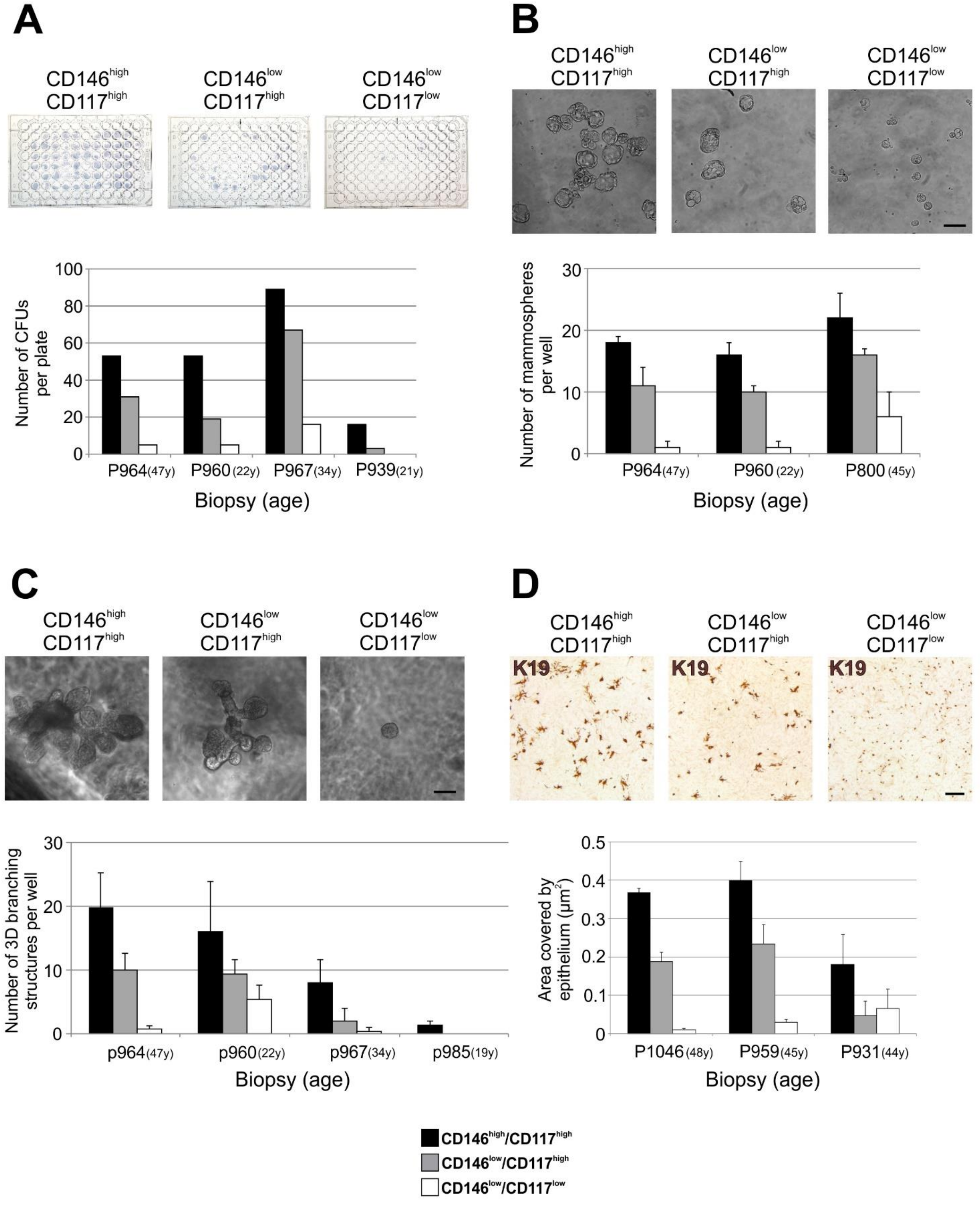
A
CD146high CD117high
CD146low CD117high
CD146low CD117low
Number of CFUs per plate
P964(47y)
P960(22y)
P967(34y)
P939(21y)
Biopsy (age)
B
Number of mammospheres per well
P800(45y)
C
Number of 3D branching structures per well
p964(47y)
p960(22y)
p967(34y)
p985(19y)
D
K19
Area covered by epithelium (µm2)
P1046(48y)
P959(45y)
P931(44y)
CD146high/CD117high
CD146low/CD117high
CD146low/CD117low

**Supplementary Figure S3**. CD146$^{high}$/CD117$^{high}$, CD146$^{low}$/CD117$^{high}$ and CD146$^{low}$/CD117$^{low}$ cells differ in functional assays of progenitor activity. For each assay, representative images of cells/structures are shown along with bar diagrams of quantitative data (CD146$^{high}$/CD117$^{high}$ (black bars), CD146$^{low}$/CD117$^{high}$ (grey bars), CD146$^{low}$/CD117$^{low}$ (white bars)) from the biopsies tested. The graphs detail the data presented collectively in Figure 3B. (A) Colony forming unit (CFU) assay with directly sorting 10 cells per well with into one 96 well plate per population. (B) Mammosphere assay with 5,000 cells per well in triplicate for each population. Bar, 100 µm. (C) 3D morphogenesis assay with 20,000 cells per well in triplicate for each population inoculated in Matrigel. Bar, 100 µm. (D) Branching morphogenesis upon epithelial-stromal interaction with 5,000 cells per flask seeded onto CD105$^{high}$ fibroblast feeders in triplicate. Bar, 250 µm. Irrespective of the biopsy tested, the CD146$^{high}$/CD117$^{high}$cells exhibit the highest progenitor cell activity. Error bars indicate SDs.

## Supplementary tables

**Supplementary Table S1.** Quantification of the presence of K14$^+$ luminal cells in 40 normal breast gland structures, divided into lobules and ducts. Structures were evaluated based on immunoperoxidase stainings of tissue sections. A structure was determined K14$^+$ when one or more luminal cells within the structure were identified as positive. Data are included in Figure 1 and Figure 4B.

| No. | Biopsy ID | Age (years) | Structural location | Total structures | K14$^+$ | % positive |
|---|---|---|---|---|---|---|
| 1 | P648 | 13 | lobules | 11 | 0 | 0 |
| 2 | W430/3 | 15 | lobules | 3 | 0 | 0 |
| 3 | W428/1 | 17 | lobules | 27 | 0 | 0 |
| 4 | W430/1 | 17 | lobules | 10 | 0 | 0 |
| 5 | W466/3 | 18 | lobules | 9 | 0 | 0 |
| 6 | P790 | 18 | lobules | 9 | 0 | 0 |
| 7 | P944 | 18 | lobules | 13 | 1 | 7.7 |
| 8 | P1094 | 18 | lobules | 13 | 0 | 0 |
| 9 | F526 | 19 | lobules | 6 | 0 | 0 |
| 10 | W403/2 | 19 | lobules | 14 | 0 | 0 |
| 11 | P816 | 20 | lobules | 35 | 1 | 2.9 |
| 12 | P652 | 20 | lobules | 5 | 0 | 0 |
| 13 | W562/1 | 21 | lobules | 6 | 0 | 0 |
| 14 | W458/1 | 22 | lobules | 5 | 0 | 0 |
| 15 | W1908/3 | 23 | lobules | 11 | 0 | 0 |
| 16 | F518 | 24 | lobules | 10 | 1 | 10 |
| 17 | P644 | 25 | lobules | 11 | 0 | 0 |
| 18 | W1916/6 | 27 | lobules | 10 | 1 | 10 |
| 19 | P645 | 29 | lobules | 10 | 2 | 20 |
| 20 | P820 | 29 | lobules | 12 | 1 | 8.3 |
| 21 | P672 | 30 | lobules | 8 | 1 | 12.5 |
| 22 | P832 | 32 | lobules | 12 | 8 | 66.7 |
| 23 | P683 | 33 | lobules | 15 | 2 | 13.3 |
| 24 | P636 | 34 | lobules | 20 | 1 | 5.0 |
| 25 | P671 | 36 | lobules | 43 | 13 | 30.2 |
| 26 | P809 | 39 | lobules | 9 | 7 | 77.8 |
| 27 | P653 | 41 | lobules | 8 | 0 | 0 |
| 28 | P880 | 43 | lobules | 7 | 1 | 14.3 |
| 29 | W901 | 43 | lobules | 15 | 3 | 20 |
| 30 | P659 | 43 | lobules | 9 | 0 | 0 |
| 31 | P819 | 45 | lobules | 12 | 2 | 16.7 |
| 32 | F498 | 46 | lobules | 20 | 2 | 10 |
| 33 | W438 | 49 | lobules | 35 | 12 | 34.3 |
| 34 | P637 | 49 | lobules | 24 | 5 | 20.8 |
| 35 | W371/3 | 52 | lobules | 19 | 1 | 5.3 |
| 36 | P634 | 56 | lobules | 10 | 5 | 50 |
| 37 | P647 | 56 | lobules | 7 | 3 | 42.9 |
| 38 | P940 | 59 | lobules | 6 | 0 | 0 |

| | | | | | | |
|---|---|---|---|---|---|---|
| 39 | W430/2 | 59 | lobules | 16 | 0 | 0 |
| 40 | P828 | 74 | lobules | 7 | 0 | 0 |
| 1 | P648 | 13 | ducts | 6 | 3 | 50 |
| 2 | W430/3 | 15 | ducts | 3 | 3 | 100 |
| 3 | W428/1 | 17 | ducts | 8 | 2 | 25 |
| 4 | W430/1 | 17 | ducts | 11 | 6 | 54.5 |
| 5 | W466/3 | 18 | ducts | 9 | 5 | 55.6 |
| 6 | P790 | 18 | ducts | 6 | 3 | 50 |
| 7 | P944 | 18 | ducts | 6 | 3 | 50 |
| 8 | P1094 | 18 | ducts | 10 | 3 | 30 |
| 9 | F526 | 19 | ducts | 5 | 3 | 60 |
| 10 | W403/2 | 19 | ducts | 9 | 2 | 22.2 |
| 11 | P816 | 20 | ducts | 8 | 3 | 37.5 |
| 12 | P652 | 20 | ducts | 6 | 1 | 16.7 |
| 13 | W562/1 | 21 | ducts | 6 | 1 | 16.7 |
| 14 | W458/1 | 22 | ducts | 14 | 6 | 42.9 |
| 15 | W1908/3 | 23 | ducts | 8 | 4 | 50 |
| 16 | F518 | 24 | ducts | 6 | 1 | 16.7 |
| 17 | P644 | 25 | ducts | 12 | 4 | 33.3 |
| 18 | W1916/6 | 27 | ducts | 6 | 5 | 83.3 |
| 19 | P645 | 29 | ducts | 4 | 2 | 50 |
| 20 | P820 | 29 | ducts | 11 | 6 | 54.5 |
| 21 | P672 | 30 | ducts | 7 | 2 | 28.6 |
| 22 | P832 | 32 | ducts | 7 | 5 | 71.4 |
| 23 | P683 | 33 | ducts | 9 | 6 | 66.7 |
| 24 | P636 | 34 | ducts | 17 | 10 | 58.8 |
| 25 | P671 | 36 | ducts | 21 | 12 | 57.1 |
| 26 | P809 | 39 | ducts | 7 | 7 | 100 |
| 27 | P653 | 41 | ducts | 5 | 1 | 20 |
| 28 | P880 | 43 | ducts | 6 | 4 | 66.7 |
| 29 | W901 | 43 | ducts | 9 | 5 | 55.6 |
| 30 | P659 | 43 | ducts | 8 | 6 | 75.0 |
| 31 | P819 | 45 | ducts | 8 | 6 | 75.0 |
| 32 | F498 | 46 | ducts | 8 | 4 | 50 |
| 33 | W438 | 49 | ducts | 14 | 11 | 78.6 |
| 34 | P637 | 49 | ducts | 10 | 7 | 70.0 |
| 35 | W371/3 | 52 | ducts | 14 | 13 | 92.9 |
| 36 | P634 | 56 | ducts | 9 | 6 | 66.7 |
| 37 | P647 | 56 | ducts | 6 | 6 | 100 |
| 38 | P940 | 59 | ducts | 7 | 5 | 71.4 |
| 39 | W430/2 | 59 | ducts | 9 | 1 | 11.1 |
| 40 | P828 | 74 | ducts | 8 | 4 | 50 |

**Supplementary Table S2**. Quantification of $K14^+$ DP luminal cells in normal breast gland structures stained by double-labeling immunofluorescence with general luminal keratin marker CAM5.2 against K7/8. The number of unique structures evaluated for each biopsy is shown in parenthesis. In all 5 biopsies a higher proportion of DP cells was present in ducts.

| No. | Biopsy ID | Age (years) | Structures | $K14^+$/total $K7/8^+$ |
|---|---|---|---|---|
| 1 | P644 | 25 | Lobules (5) | 0/518 (0.0%) |
| | | | Ducts (7) | 26/469 (5.5%) |
| 2 | P832 | 32 | Lobules (5) | 10/770 (1.3%) |
| | | | Ducts (5) | 17/377 (4.5%) |
| 3 | P683 | 33 | Lobules (7) | 1/604 (0.2%) |
| | | | Ducts (3) | 66/407 (16.2%) |
| 4 | P809 | 39 | Lobules (4) | 117/706 (16.6%) |
| | | | Ducts (5) | 187/609 (30.7%) |
| 5 | W438 | 46 | Lobules (4) | 35/606 (5.8%) |
| | | | Ducts (4) | 82/422 (19.4%) |

**Supplementary Table S3.** Topological evaluation of CD146 expression in luminal cells in the normal breast gland by immunohistochemical staining of 20 biopsies. -, +, ++ and +++ are indications of the relative frequency of $CD146^{+}$ luminal cells. In 19 out of 20 biopsies, a more prominent CD146 expression was observed in ducts. * p< 0.00001, tested by Mann-Whitney test.

| **No.** | **Biopsy ID** | **Age (years)** | **Reaction in ducts*** | **Reaction in lobules** | **Most prominent in ducts** |
|---|---|---|---|---|---|
| 1 | P648 | 13 | ++ | + | • |
| 2 | W430/1 | 17 | ++ | + | • |
| 3 | W445 | 20 | ++ | + | • |
| 4 | P652 | 20 | +++ | - | • |
| 5 | P722 | 20 | - | - | |
| 6 | W1908/3 | 23 | + | - | • |
| 7 | P663 | 24 | ++ | + | • |
| 8 | P644 | 25 | +++ | - | • |
| 9 | P820 | 29 | ++ | + | • |
| 10 | P672 | 30 | ++ | + | • |
| 11 | P683 | 33 | + | - | • |
| 12 | P772 | 33 | ++ | - | • |
| 13 | P636 | 34 | +++ | + | • |
| 14 | P671 | 36 | +++ | + | • |
| 15 | P653 | 40 | +++ | ++ | • |
| 16 | P633 | 45 | +++ | + | • |
| 17 | F498 | 46 | ++ | + | • |
| 18 | W652 | 46 | + | - | • |
| 19 | P637 | 49 | ++ | + | • |
| 20 | P647 | 56 | + | - | • |

**Supplementary Table S4.** Relative distribution of $CD117^+$ and $CD146^+$ luminal cells in ducts and lobules from normal breast tissue in serial immunostained sections. +, ++ and +++ are indications of the relative frequency of $CD117^+$ cells compared to $CD146^+$ cells. In all eight biopsies a higher frequency of $CD117^+$ cells was observed in ducts and lobules. * p< 0.01, tested by Mann-Whitney test.

| No. | Biopsy ID | Age (years) | Ducts, CD117/CD146 ratio* | Lobules, CD117/CD146 ratio |
|---|---|---|---|---|
| 1 | P652 | 20 | + | +++ |
| 2 | P820 | 29 | ++ | +++ |
| 3 | P683 | 33 | ++ | +++ |
| 4 | P772 | 33 | ++ | +++ |
| 5 | P671 | 36 | ++ | ++ |
| 6 | P653 | 40 | + | ++ |
| 7 | P633 | 45 | ++ | +++ |
| 8 | F498 | 46 | +++ | +++ |

Supplementary Tables S5-S8 are available as individual excel documents:

**Supplementary Table S5.** Summary of RNA sequencing data from $CD146^{high}/CD117^{high}$ (CD146), $CD146^{low}/CD117^{high}$ (CD117) and $CD146^{low}/CD117^{low}$ (CD326) luminal populations from 3 biopsies (P960, P964 and P967) (mapping to reference genome).

**Supplementary Table S6**. RNA-seq data analysis of $CD146^{high}/CD117^{high}$ (CD146), $CD146^{low}/CD117^{high}$ (CD117) and $CD146^{low}/CD117^{low}$ (CD326) luminal populations from 3 biopsies (P960, P964 and P967).

**Supplementary Table S7**. List of differently expressed genes by NOISeq comparing $CD146^{high}/CD117^{high}$ vs $CD146^{low}/CD117^{high}$, $CD146^{low}/CD117^{low}$ vs $CD146^{high}/CD117^{high}$ and $CD146^{low}/CD117^{low}$ vs $CD146^{low}/CD117^{high}$ populations.

**Supplementary Table S8.** Supplementary Table S8. Summary of gene ontology analysis of DEGs. A-C: Comparison of $CD146^{high}/CD117^{high}$ (CD146) and $CD146^{low}/CD117^{high}$ (CD117). D-F: Comparison of CD117 and $CD146^{low}/CD117^{low}$ (CD326). G-I: Comparison of CD146 and CD326.

**Supplementary Table S9.** Quantification of the frequency of DP cells in smears of trypsinized primary breast tissue. Twenty biopsies were stained for K14/K19, and seven of these were additionally stained for K14/K7/8. The data are included in Figure 5A.

| **No.** | **Biopsy ID** | **Age** | **$K14^+$/total $K19^+$** | **$K14^+$/total $K7/8^+$** |
|---|---|---|---|---|
| 1 | P985 | 19 years | 7/831 (0.8%) | |
| 2 | P790 | 19 years | 11/620 (1.8%) | 7/633 (1.1%) |
| 3 | P722 | 20 years | 27/600 (4.5%) | |
| 4 | P939 | 21 years | 6/508 (1.2%) | |
| 5 | P960 | 22 years | 58/1040 (5.6%) | 59/1050 (5.6%) |
| 6 | P987 | 23 years | 33/1020 (3.2%) | |
| 7 | P848 | 23 years | 23/515 (4.5%) | 19/532 (3.6%) |
| 8 | P749 | 25 years | 24/571 (4.2%) | 13/572 (2.3%) |
| 9 | P636 | 34 years | 44/1000 (4.4%) | |
| 10 | P967 | 34 years | 298/919 (32.4%) | |
| 11 | P640 | 35 years | 21/596 (3.4%) | |
| 12 | P952 | 37 years | 22/500 (4.4%) | |
| 13 | P966 | 41 years | 68/1062 (6.4%) | 70/1029 (6.8%) |
| 14 | P653 | 41 years | 40/360 (11.1%) | |
| 15 | P727 | 44 years | 25/570 (4.4%) | 16/560 (2.9%) |
| 16 | P737 | 44 years | 78/939 (8.3%) | 49/974 (5.0%) |
| 17 | P964 | 47 years | 92/579 (15.9%) | |
| 18 | P936 | 54 years | 20/483 (4.1%) | |
| 19 | P937 | 54 years | 81/562 (14.4%) | |
| 20 | P962 | 59 years | 38/437 (8.7%) | |

**Supplementary Table S10.** An overview of the presence of vDP cells in lobules and ducts of women with no known *BRCA1* mutations (normal) and of *BRCA1* mutation carriers. The predominant phenotype is marked with '•' in lobules and ducts for each biopsy. In cases where both phenotypes were found in equal amounts, both are marked. Cases where no vDP/DP cells were identified are marked with '-'. In general, lobules contain vDP cells while ducts have DP cells co-expressing CD146. For *BRCA1* mutation carriers age was not available (na).

| | | | Lobules | | Ducts | |
|---|---|---|---|---|---|---|
| | | Age | vDP cells without CD146 | DP cells with CD146 | vDP cells without CD146 | DP cells with CD146 |
| **Normal** | | | | | | |
| | W430/1 | 17 | - | - | | • |
| | W466/3 | 18 | - | - | | • |
| | P790/1 | 18 | - | - | | • |
| | P944 | 18 | - | - | | • |
| | W458/1 | 22 | - | - | | • |
| | F518 | 24 | • | | | • |
| | P644 | 25 | - | - | | • |
| | W1916/6 | 27 | - | - | | • |
| | P832 | 32 | • | | | • |
| | P636 | 34 | - | - | | • |
| | P671 | 36 | • | | • | |
| | P809 | 39 | • | | | • |
| | W901 | 43 | • | | | • |
| | W438 | 46 | | • | | • |
| | P637 | 49 | • | | • | |
| | P634 | 56 | • | | • | • |
| **BRCA1 mutation carriers** | | | | | | |
| | P705 | na | - | - | | • |
| | P714 | na | • | | | • |
| | P728 | na | • | • | | • |
| | P734 | na | • | | | • |
| | P742 | na | • | | | • |
| | P744 | na | • | | • | |
| | P754 | na | • | | | • |
| | P825 | na | • | | | • |
| | P847 | na | • | | | • |
| | P911 | na | - | - | | • |

**Supplementary Table S11.** Quantification of the frequency of DP cells expressing Ki67 evaluated by immunofluorescence on tissue sections from normal women (normal) and *BRCA1* mutation carriers (BRCA1). It should be noted that the biopsies listed as normal are from women presumed not to have a BRCA1 mutation, as they were not tested. The average age of donor of normal breast biopsies was 44.6 years. For *BRCA1* mutation carriers information on age was not available (na). The data are included in Figure 6.

| Status | Biopsy ID | Age (years) | $Ki67^{+}$-DP/total DP cells | % |
|---|---|---|---|---|
| normal | P647 | 56 | 0/174 | 0% |
| normal | W438 | 46 | 2/511 | 0.4% |
| normal | P832 | 32 | 0/273 | 0% |
| normal | P634 | 56 | 0/355 | 0% |
| normal | P671 | 36 | 10/439 | 2.3% |
| normal | P880 | 43 | 1/322 | 0.3% |
| normal | W901 | 43 | 0/283 | 0% |
| BRCA1 mutated | P714 | na | 11/167 | 6.6% |
| BRCA1 mutated | P734 | na | 4/221 | 1.8% |
| BRCA1 mutated | P744 | na | 6/544 | 1.1% |
| BRCA1 mutated | P754 | na | 8/443 | 1.8% |
| BRCA1 mutated | P825 | na | 4/252 | 1.6% |
| BRCA1 mutated | P847 | na | 1/398 | 0.3% |
| BRCA1 mutated | P852 | na | 3/124 | 2.4% |

**Supplementary Table S12**. Overview of the CD146 status of tumors that are predominantly DP positive. Six carcinomas with >50% of DP neoplastic cells were selected from 23 ERα-negative tumors expressing K14. Five of the six tumors did not express CD146.

| **Tumor** | | | vDP cells without CD146 | DP cells with CD146 |
|---|---|---|---|---|
| 1 | P860 | - | ● | |
| 2 | P914 | - | ● | |
| 3 | P942 | - | ● | |
| 4 | P994 | - | ● | |
| 5 | P1049 | - | ● | |
| 6 | W1884/3 | - | | ● |

**Supplementary Table S13**. List of antibodies used for immunostaining/FACS.

| Antibody | Clone | Company/Cat. no. | Peroxidase | Fluorescence | FACS |
|---|---|---|---|---|---|
| α-smooth muscle actin | 1A4 | Sigma, A5691 | - | 1:500 | - |
| CD117 | K45 | NeoMarkers, MS-289 | 1:100 | 1:50 | - |
| CD117, PE | 104D2 | BD Biosciences, 332785 | - | - | 1:20 |
| CD146 | P1H12 | Abcam, ab24577 | 1:1000 | 1:100 | - |
| CD146, AF647 | P1H12 | BD Biosciences, 563619 | - | - | 1:20 |
| CD326, PerCP/Cy5.5 | 9C4 | BioLegend, 324214 | - | - | 1:20 |
| CD326, BV421 | EBA-1 | BD Biosciences, 563180 | - | - | 1:20 |
| ERα | 1D5 | Dako, M7047 | 1:100 | - | - |
| Keratin 5 | XM26 | Novocastra, NCL-L-CK5 | - | 1:100 | - |
| Keratin 5 | - | Covance, PRB-160P | - | 1:100 | - |
| Keratin 7/8 | CAM5.2 | BD Biosciences, 345779 | - | 1:10-1:25 | - |
| Keratin 14 | LL002 | Monosan, | 1:100 | 1:25 | |

| | | | | | |
|---|---|---|---|---|---|
| | | MONX10687 | | | |
| Keratin 15 | LHK15 | NeoMarkers, MS-1068 | - | 1:50 | - |
| Keratin 17 | E3 | Dako, M7046 | 1:100 | 1:25 | - |
| Keratin 19 | A53-B/A2 | BioLegend, 628502 | - | 1:25 | - |
| Keratin 19 | BA16 | GenWay, GWBC266E | 1:200 | - | - |
| Keratin Ks20.8 | Ks20.8 | Dako, M7019 | - | 1:10 | - |
| Ki-67 | MIB-1 | Dako, M7240 | - | 1:25 | - |
| SSEA4 | MC813-70 | Millipore, MAB4304 | - | 1:25 | - |
| Alexa Fluor 488 goat anti mouse IgG1 | - | Invitrogen, A21121 | - | 1:500 | - |
| Alexa Fluor 488 goat anti mouse IgG3 | - | Invitrogen, A21151 | - | 1:500 | - |
| Alexa Fluor 568 goat anti mouse IgG1 | - | Invitrogen, A21124 | - | 1:500 | - |
| Alexa Fluor 568 goat anti mouse IgG2a | - | Invitrogen, A21134 | - | 1:500 | - |

| | | | | | |
|---|---|---|---|---|---|
| Alexa Fluor 568 goat anti mouse IgG2b | - | Invitrogen, A21144 | - | 1:500 | - |
| Alexa Fluor 568 goat anti-rabbit IgG | - | Molecular Probes, A-11036 | - | 1:500 | - |
| Alexa Fluor 647 goat anti-rabbit IgG2a | - | Invitrogen, A21241 | - | 1:500 | - |
| Rhodamine Red goat anti-mouse IgG3 | - | Jackson ImmunoResearch, 115-295-209 | - | 1:100 | - |
